# Comparison of Skin Reaction between MTA (Produced in Iran) and CEM in Rabbit

**Published:** 2012

**Authors:** Marziyeh Shokravi, Bahareh Tabarsi, Aliakbar Moghaddamnia, Alieh Sohanfaraji, Mohsen Pourghasem

**Affiliations:** 1 *Cellular and Molecular Biology Research Center, Babol University of Medical Sciences, Babol, Iran. *; 2 *School of Dentistry, Babol University of Medical Sciences, Babol, Iran. *; 3 *Department of Pharmacology, Babol University of Medical Sciences, Babol, Iran. *

**Keywords:** Endodontics, skin reaction, MTA, CEM

## Abstract

Pathological changes in pulp and periapical tissues are addressed by endodontic treatment. The material used in this treatment must be biocompatible. The aim of this study is to compare the skin reaction of Calcium Enriched Mixture (CEM) and Mineral Trioxide Aggregate (MTA) produced in Iran on rabbit.

Sixteen male newzeland albino rabbits weighting 2 kg were used. The animals back hair was shaved, 24 hours before application of each material. The material was applied on two sites (2 × 2 cm) while the third site was used as control. All sites were covered by gauze and bandaged for 4 hours. Then the material's remnants were washed off the sites of application. Observations were performed in 1, 24, 48 and 72 hours after removing the materials. Erythematous surface areas were measured by the morphometric method. After sacrificing animals the skins were dissected and the specimens were prepared for histological evaluation.

There were significant differences between CEM and MTA in erythematous surface areas at 1, 24 and 48 hours after removing the materials (p<0.05). However there was no significant difference at 72 hours after removing the materials.

Data showed significant differences in counted cells between MTA and control sample (p=0.0001) and between MTA and CEM (p=0.035). There was no significant difference between control and CEM (p>0.05).

The average erythematous surface areas were wider in MTA sites than CEM sites. As a conclusion it seems that biocompatibility of CEM could be more than MTA.

Surgical endodontic therapy is one of the well-known methods to repair problems of root canal systems in some cases (1). In this method, a suitable root-end filling material may be applied into the prepared root-end. Dental filling materials seal the root canal system and should stick to the preparation walls. Unique properties of these materials including: non-toxic, non- absorbable, non-carcinogenic, well toleration by periradicular tissues, speed up healing should be considered. Moreover, materials should be dimensionally stable and good tolerated against moisture. In addition, easy to manipulate and be radiopaque are desirable (2).

In 1993, original Mineral Trioxide Aggregate (MTA) was introduced by Torabinejad et al. at Loma University (3). It is a powder consisting of five types of hydrophilic particles which could be set in the presence of moisture. MTA is a mixture of calcium silicate (CaSio_4_), bismuth oxide (Bi2O3), calcium carbonate (CaCo3), calcium sulphate (CaSo4) and calcium aluminate (CaAl2o4). It is used for direct pulp capping, repairing of root perforation, apexification and root end filling (4-5). It is not necessary to stop bleeding completely prior placing the MTA. Apparently, MTA would not be deteriorated by time; therefore, there is no space for micro leakage (6). However, it has some disadvantages including a delayed setting time, poor handling characteristics and high price (5, 7-8)

Recently a new endodontic material that is named CEM (Calcium Enriched Mixture) has been introduced (7). It consists of different calcium compound calcium Oxide, Calcium Phosphate, Calcium Carbonate, Calcium Silicate, Calcium Sulphate, Calcium Hydroxide and Calcium Chloride. In contrast with MTA, CEM has better setting time (less than 1 hour), handling characteristics, chemical properties, higher flow rate, less film thickness, and a reasonable price (7-8). This cement forms an effective seal against microorganism, has an antibacterial effect and is resistant to wash out and able to set in an aqueous environment. CEM is also able to produce hydroxyapatite (7, 9). Antibacterial effects of CEM are comparable with MTA (10). An ideal root-end filling material should have well histocompatiblity property. This study aimed to compare skin reaction of MTA and CEM on rabbit.

## Materials and Methods

Sixteen male Newzeland albino rabbits weighting 2kg (Pasteur institute, Tehran-Iran) were used in this study. Thes animals were housed singly with standard laboratory condition, 12 hours light/darkness cycle, constant temperature, 50-55% moisture and easy access to food and water. Animal care was performed in according with Ethical Committee of Babol University of Medical Sciences. As a pilot, first evaluation was performed on one rabbit, and then study was continued by other animals. Animals back fur were shaved gently 24 hours before application of materials. Clipped area of each rabbit was divided into 3 equal sites (2 × 2 cm). Back of animals were disinfected with povidon-iodine (Betadin 10%) and washed out 24 hours later. Then MTA and CEM were used. To apply materials, a wax strip (10 × 10 cm^2^) with three retro angular sites which matched by clipped area was used . In each rabbit one site was used as control and two sites were experimental sites. To provide a sandy mixture MTA was mixed according to manufactured instruction. CEM was also mixed by its liquid  to provide a dense creamy mixture . Then each material was placed in one experimental site of each animal. 

For induction of spatulation effect, spatula was rubbed with distilled water, then both treated and control sites were covered by gauze and bandaged. After 4 hours, the gauze and bandage were removed and the materials were washed out. Observations were performed in 1, 24, 48, and 72 hours. A transparency paper was used to mark reaction areas of back of animals. Then Cavalier’s method was used to calculate area of reactions (11). In this method a spotted paper with distance 3 mm between dots was applied. Each dot was consisted equally a rectangular area with 9 mm^3^. To analyse the data t-test was performed.

After 72 hours animals were sacrificed and skin specimens was collected and placed in 10% formalin solution for 48 hours. Five micron slices were prepared and stained by H & E for histological purposes. The sections were blindly examined by another observer. For each sample 5 slides with 7 sections were prepared and observed under a light microscope (Olympus ×400). Quantitation of inflammatory cells in histopathology slides was performed. In two slides 10 separated areas of 10 sections were chosen randomly and the images transferred to computer by camera. Finally, a round area with 6 cm diameter was detected on the other transparency paper and the number of inflammatory cells in that area was counted and average of values for each material was calculated. Data was analysed with ANOVA test.

## Results

**Fig 1:**
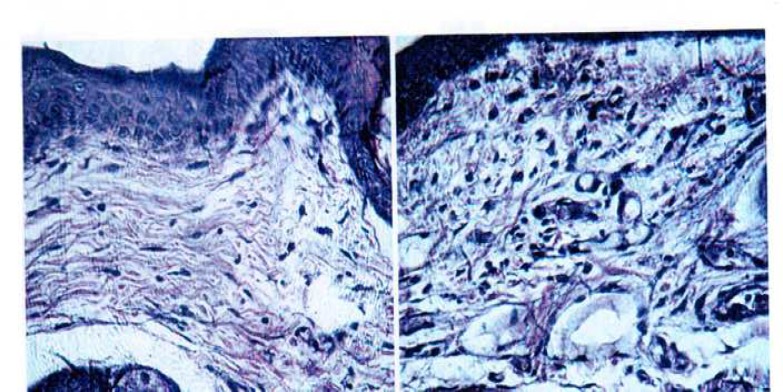
Cellular infiltration. Cells increased in dermis of MTA sample (left picture). X400

Findings of this study show that rabbits’ skin reactions to MTA were more than CEM. Dilatation of the vessels was  observed in dermis of CEM treated skin samples. But this dilatation was more sever in MTA samples. There was little increasing in number of cells in dermis of CEM compared to control group. However, infiltration of cells in dermis of MTA samples clearly increased compared to CEM samples (Fig.1 and Table 1).

**Table 1:** Counts of infiltrated cells 72 h after washing 2 different sealers (MTA and CEM) off the rabbits skin

Control	CEM	MTA
Mean	5.07	8.13
SD	1.51	2.53

**Fig 2:**
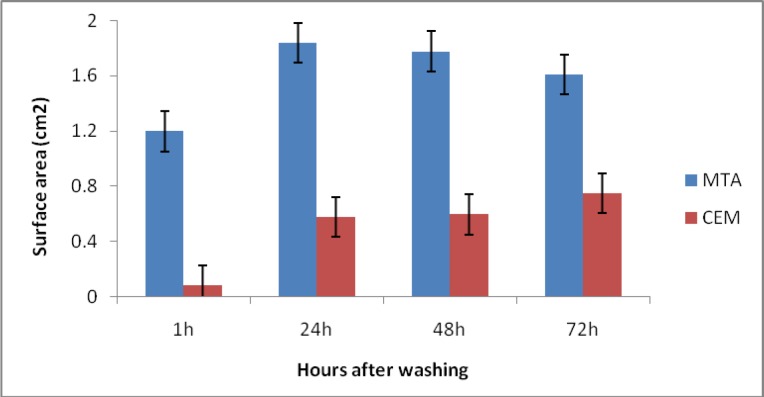
Comparison of skin reactin induced by MTA and CEM by time

Regarding to skin reaction, there were significant differences between CEM and MTA samples in all times (P<0.05) (Fig.2).

ANOVA analyses showed significant differences in counted cells between control and MTA samples (P= 0.0001) and also between MTA and CEM samples (Table 1). However there were no significant differences between control and CEM samples (P>0.05). 

## Discussion

As mentioned in the introduction, MTA has a delayed setting time, poor handling characteristics and a high price. Because of these MTA disadvantages, CEM as a new experimental cement has been introduced. The aim of this study was comparison of the biocompatibility  of these two materials. Skin irritation test is a simple method that could be as first step of biocompatibility evaluation. This study compares the skin reactions of induced by CEM and MTA which its results show that more severe reaction occurs with MTA. There were significant differences in skin reactions induced by MTA and CEM at 1, 24, 48, and 72 hours after washing off the materials from the animals skin. Histopathological evaluation of the skin samples revealed that the number of inflammatory cells had increased in MTA samples compared with CEM samples. Results of this study are comparable with previous studies. Moretton et al. reported that MTA induces sever reaction with coagulation necrosis by subset of inflammation with time (12). Yaltrik also found that MTA causes inflammatory response which reduced after 60 and 90 days (13). Sumer et al. showed that MTA induces an initial sever inflammatory reaction that subsided during 60 days (14). The results of this study were different from some studies which reported a low reaction.

MTA has pH 10.2 immediately after mixing and reach to 12.5 after 3 hours . After 168 hours the pH decreases to 9.5 (15). Therefore High pH is expected especially when the materials are freshly mixed. It causes denaturation of adjacent cells and tissue protein and after setting of materials the pH changes and injures subside (16).

The initial inflammatory response may be caused by calcium hydroxide (CaOH2) which is produced by mixing MTA powder with water. The CaOH2 reacts with tissue fluid and produces hydroxyapatite crystals that cover the surface of MTA which consequently subsides the inflammation (17).

High alkalinity of CEM is comparable with white MTA. In addition to the presence of CaOH2 in CEM, during and after mixing of CEM with its setting solution (PBS), hydration reaction takes place and produces more CaOH2. CaOH2 would be dissociated into Ca^2+^ and hydroxyl ions which rise the pH (8, 9).

In this study the setting solutions of two materials were not similar. According to manufacturer direction, MTA was mixed with distilled water and CEM was mixed with PBS (7). Study of Lotfi et al. showed that MTA mixed with disodium hydrogen phosphate causes mild inflammation after 7 days (18). MTA makes a moderate inflammatory reaction alone. We hypothesise that less inflammatory reaction of CEM than MTA could be explained by the presence of disodium hydrogen phosphate. One study showed that hydroxyapatite crystal has been produced upon MTA and CEM contact with PBS solution  which contained phosphate ions. Surface topography of CEM samples which were immersed in normal saline solution was altered with white crystal formation, but this process was not found in MTA samples (19). It is hypothesised that CEM has endogenous source of calcium and phosphate ions.

We expect more severe reaction when endodontic materials are placed adjacent to pulp and periradicular tissues. As a conclusion, it seems that biocompatibility of CEM is better than MTA at least in short term.
